# LncRNA MALAT1 aggravates oxygen‐glucose deprivation/reoxygenation-induced neuronal endoplasmic reticulum stress and apoptosis via the miR-195a-5p/HMGA1 axis

**DOI:** 10.1186/s40659-021-00331-9

**Published:** 2021-03-09

**Authors:** Ying Jia, Lian Yi, Qianqian Li, Tingjiao Liu, Shanshan Yang

**Affiliations:** Department of Neurology, The First Affiliated Hospital of Harbin Medical University, No.23 Youzheng Street, Nangang District, Harbin, 150081 Heilongjiang People’s Republic of China

**Keywords:** Ischemic stroke, MALAT1, Oxygen‐glucose deprivation/reoxygenation, Neuronal injury, Endoplasmic reticulum stress

## Abstract

**Background:**

This study aimed to investigate the potential role and molecular mechanism of lncRNA metastasis associated lung adenocarcinoma transcript 1 (MALAT1) in cerebral ischemia/reperfusion injury.

**Results:**

Using an oxygen-glucose deprivation/reoxygenation (OGD/R) cell model, we determined that the expression of MALAT1 was significantly increased during OGD/R. MALAT1 knockdown reversed OGD/R-induced apoptosis and ER stress. Mechanistically, MALAT1 promoted OGD/R-induced neuronal injury through sponging miR-195a-5p to upregulating high mobility group AT-hook1 (HMGA1).

**Conclusions:**

Collectively, these data demonstrate the mechanism underlying the invovlvement of MALAT1 in cerebral ischemia/reperfusion injury, thus providing translational evidence that MALAT1 may serve as a novel biomarker and therapeutic target for ischemic stroke.

## Background

Stroke is a common cerebrovascular disease and the third leading cause of death in China, with 1.57 million deaths in 2018 [1]. Ischemic stroke is by far the most common type of stroke, accounting for about 87 % of all strokes [2]. Thrombolysis therapy is the most effective strategy to restore cerebral blood flow timely for ischemic stroke patients [3]. However, blood flow restoration not only improves the oxygen supply but also induces the overproduction of ROS, activates inflammation and immune response, and ultimately triggers cell death programs [4]. Therefore, a comprehensive understanding of the mechanisms underlying neuronal death during cerebral ischemia/reperfusion (I/R) injury is crucial to the development of neuroprotective therapies and the rescue of injured neurons in the brain.

Endoplasmic reticulum (ER) stress pathway is an important apoptotic pathway and has been reported to induce apoptosis in numerous diseases including metabolic, neurodegenerative and cardiovascular diseases, cancer, inflammation, and viral infections [5]. ER is a dynamic organelle in eukaryotic cells and participates in protein synthesis, modification and processing, folding, assembly, and the transportation of nascent peptide chains. Under stressful situations, compromised ER environment and impaired protein maturation cause the misfolded or unfolded proteins to accumulate and activate the ER stress signal that transmits to the nucleus through the ER membrane. Transient and slight ER stress contributes to maintain cellular homeostasis. However, prolonged activation of ER stress triggers cell death [6]. Studies have confirmed a close association between activation of ER stress pathway and cerebral ischemia reperfusion injury [7]. In this study, two key proteins, glucose-regulated protein 78 (GRP78) and C/EBP homologous protein (CHOP) in ER stress pathway were selected to examine the ER stress.

Long noncoding RNAs (LncRNAs) are transcripts more than 200 nucleotides that regulate gene expression at the levels of chromatin modification, transcriptional and post-transcriptional processing [8]. LncRNA MALAT1 was originally identified in non-small cell lung cancer and associated with metastasis in lung adenocarcinoma [9]. Subsequent studies showed that MALAT1 is aberrantly expressed in multiple cancerous tissues and associated with the proliferation and metastasis of tumor cells [10–12]. Recent studies have demonstrated that MALAT1 was implicated in ischemic stroke. Qi Gao et al. [13] found that MALAT1 was downregulated in brain microvascular endothelial cells (BMEC) in OGD condition and knockdown of MALAT1 promoted OGD-induced apoptosis. Moreover, Hongwei Wang et al. [14] revealed that MALAT1 was upregulated in middle cerebral artery occlusion (MCAO)/reperfusion model and astrocyte cell OGD/RX model, whereas inhibition of MALAT1 protected against ischemia-reperfusion-induced astrocyte cell injury via miR-145/AQP4 axis. Taken together, these findings suggest MALAT1 plays vital roles in the progression of cerebral ischemic stroke. However, the role and mechanism of MALAT1 in cerebral I/R-mediated neuronal cell death remains unclear.

MicroRNAs (miRNAs) are a class of small (18 ~ 25 nt), non-coding single-stranded RNA molecules that negatively regulate gene expression by targeting the 3′UTR regions of the genes [15]. It has been reported that miRNAs play important roles in the process of cerebral ischemia reperfusion injury [16]. For example, Yu et al. [17] reported that miR-670 aggravates cerebral ischemia/reperfusion injury via the Yap pathway. Another study also showed that miR-190 exerts neuroprotective effects against ischemic stroke through Rho/Rho-kinase pathway [18]. Moreover, several studies demonstrated that miRNAs regulate mitochondrial function and oxidative stress [19, 20]. All these studies underscore the important roles of miRNAs in the cerebral ischemia reperfusion injury. mir-195a-5p is a recently discovered miRNA associated with cell proliferation and cell division. Guo et al. [21] showed that ectopic expression of miR-195a-5p inhibits mouse medullary thymic epithelial cells proliferation by directly targeting Smad7, indicating the key role of miR-195a-5p in the age-related thymus involution. Adamowicz et al. [22] revealed that miR-195a-5p acts as a potential regulator of cell division in mouse and human cardiomyocytes and is involved in the withdrawal of the postnatal mouse heart from DNA replication and cell division. These studies imply the versatile roles of miR-195a-5p in cell proliferation, cell cycle and cell division. In this study, we found that miR-195a-5p contains potential binding sites of MALAT1 using bioinformatics tools. Thus, we further explored whether mir-195a-5p is involved in MALAT1-mediated ischemia-reperfusion injury.

In this study, we demonstrated that MALAT1 was elevated in the OGD/R model in a time-dependent manner. Knockdown of MALAT1 attenuated OGD/R-induced neuronal injury, which was linked to decreased levels of mitochondrial and ER stress-associated apoptosis molecules.

## Methods

### Cell culture

The neuronal cell line HT22 was obtained from American Type Culture Collection (ATCC, Manassas, VA, USA). HT22 cells were cultured in Dulbecco’s modified Eagle’s medium (DMEM) (Hyclone, USA) containing 10 % fetal bovine serum (Gibco, USA), 1 % penicillin/streptomycin and 2 mM glutamine. Cells were incubated in a humidified atmosphere containing 5 % CO_2_ at 37 °C.

For OGD/R model, HT22 cells were cultured in glucose-free DMEM under hypoxic conditions (1 % O_2_, 94 % N_2_ and 5 % CO_2_) at 37 °C for 4 h. After OGD, cells were incubated in complete medium under normoxic conditions (95 % air and 5 % CO_2_) for 12 h, 24 h and 48 h. Control cells were cultured in DMEM under normoxia.

### Cell transfection

The small interfering RNA (siRNA) targeting MALAT1 (si-MALAT1), negative control scrambled siRNA (si-NC), miR-195a-5p mimics, mimics control (mimics-NC), miR-195a-5p inhibitor (inh-miR-195a-5p), negative inhibitor (inh-NC), pcDNA3.1 targeting HMGA1 (pcDNA3.1-HMAGA1), pcDNA3.1 empty vector (pcDNA3.1-NC) were all purchased from GenePharma (Suzhou, China). The transfection of these sequences into HT22 cells was conducted by Lipofectamine 3000 reagent (Invitrogen) following the manufacturer’s instructions. After transfection, the original medium was replaced with fresh medium and cells were incubated for 48 hours for further analysis.

### Cell viability assay

The CCK-8 assay was used to determine cell viability. HT22 cells were seeded into 96-well plates at a density of 1 × 10^4^ cells/well. After indicated treatments, 10 µl of CCK8 reagent was added into each well. The 96-well plate was then incubated at 37 °C for 2 h in the dark. The optical density (OD) was measured using a microplate reader (Rayto RT-6000, Ruixin) at a wavelength of 450 nm.

### Quantitative real‐time RCR (qRT-PCR)

Total RNA was extracted using Trizol (Invitrogen, USA) and the concentration and quality of RNA were detected by NanoDrop. The A260/280 ratios of the RNA samples were 1.8–2.1. The reverse transcription reaction was performed using the High-Capacity RNA-to-cDNA™ Kit (Applied Biosystems, USA), and 1 µg RNA was used for a 10-µL reaction. Quantitative PCR was carried out with the ABI 7500 PCR System (Applied Biosystems, USA) using SYBR™ Green Master Mix (Invitrogen, USA). The relative expression of MALAT1 and miR-195a-5p were calculated using the 2^−ΔΔCt^ method [23]. β-actin was chosen as an internal control. PCR primers were listed in Table 1.

**Table 1:** Primers used for real-time PCR

Genes	Sequences	Length of amplicons
MALAT1	F: 5′-AAAGCAAGGTCTCCCCACAAG-3’ R: 5′-GGTCTGTGCTAGATCAAAAGGCA-3’	71 bp
HMGA1	F: 5′- AAGACCCGGAAAACCACCAC-3′ R: 5′- GCCCTCCTCTTCCTCCTTCT-3′	81 bp
β-actin	F: 5′- GGTCATCACCATTGGCA − 3′ R: 5′- GAGTTGAAGGTAGTTTCGTGGA-3′	105 bp

### Western blot

Western blot analysis was performed as previously described [24]. Cells were lysed in RIPA lysis buffer (Beyotime, China) on ice and total protein was quantified by a bicinchoninic acid assay. Equal amounts of protein samples (40 µg) were loaded onto each lane of 10 % sodium dodecyl sulfate-polyacrylamide gel electrophoresis (SDS-PAGE) and transferred to polyvinylidene fluoride (PVDF) membranes (Millipore, USA). The membranes were incubated with the primary antibodies at 4 °C overnight following blocking with 5 % non-fat milk for 1 h. Then, the membranes were incubated with secondary antibodies for 1 h. Finally, signals were visualized with enhanced chemiluminescence reagent (Bio-Rad, USA), and the intensity of band was quantified using image software (Bio-Rad, USA). The antibodies against HMGA1, cleaved-caspase-3, bax, bcl-2, CHOP, GRP78 and β-actin were purchased from Invitrogen. β-actin was chosen as an internal control.

### Flow cytometry

Cell apoptosis analysis was conducted by Annexin V-FITC detection kit (Beyotime, China). Cells were collected and re-suspended in PBS at 1 × 10^5^ cells/ml. After centrifuged at 1000 rpm for 5 min, 195 µl Annexin V-FITC binding buffer was added to resuspended cells. Then, 5 µl Annexin V-FITC was added into the cells. Later, 10 µl propidium iodide (PI) was added into the cells. After incubation at room temperature for 20 min at dark, flow cytometry (FACSCanto II; BD, USA) was used to detect the apoptosis of HT22 cells, and the data were analyzed using CELLQuest software.

### Luciferase reporter assay

The binding sites of MALAT1 to miR-195a-5p were analyzed using the bioinformatics website [25]. The MALAT1 3′UTR fragment containing the predicted miR-195a-5p binding site was cloned into the pmirGLO vector (RiboBio, Guangzhou, China) to form the reporter vector MALAT1-wild-type (MALAT1-wt). Next, we generated the MALAT1 mutant (-MALAT1-mut) by site mutation to mutate the putative binding site of miR-195a-5p in the MALAT1 3′UTR. The miR-195a-5p mimic and the vectors (MALAT1-wt or MALAT1-mut) were cotransfected into cells, and luciferase activity was detected using a dual luciferase reporter assay system. The miR-195a-5p and HMGA1 luciferase reporter assays were identical to those described above.

### RNA binding protein immunoprecipitation (RIP) assay

Magna RIP™ RNABinding Protein Immunoprecipitation kit (Merck Millipore, USA) was used to analyze the binding specificity between MALAT1 and miR-195a-5p. HT22 cells were washed with cold PBS and lysed in RIP lysis buffer at 4 °C. Cell lysate was incubated with RIP buffer containing magnetic beads conjugated to Ago2 antibody or IgG. Then, RNA-binding protein complex was cultured with protease K and isolated immunoprecipitated RNA. qRT-PCR was used to detect the levels of MALAT1 and miR-195a-5p in the precipitates and the whole cell lysate (input).

### Statistical analysis

Data were represented as mean ± standard error of the mean (SEM). The differences were statistically analyzed by student t test or one-way analysis of variance (ANOVA). All experiments were repeated at least three times. p value < 0.05 was considered statistically significant.

## Results

### LncRNA MALAT1 was upregulated in OGD/R-induced neuronal injury of HT22 cells

**Fig. 1:**
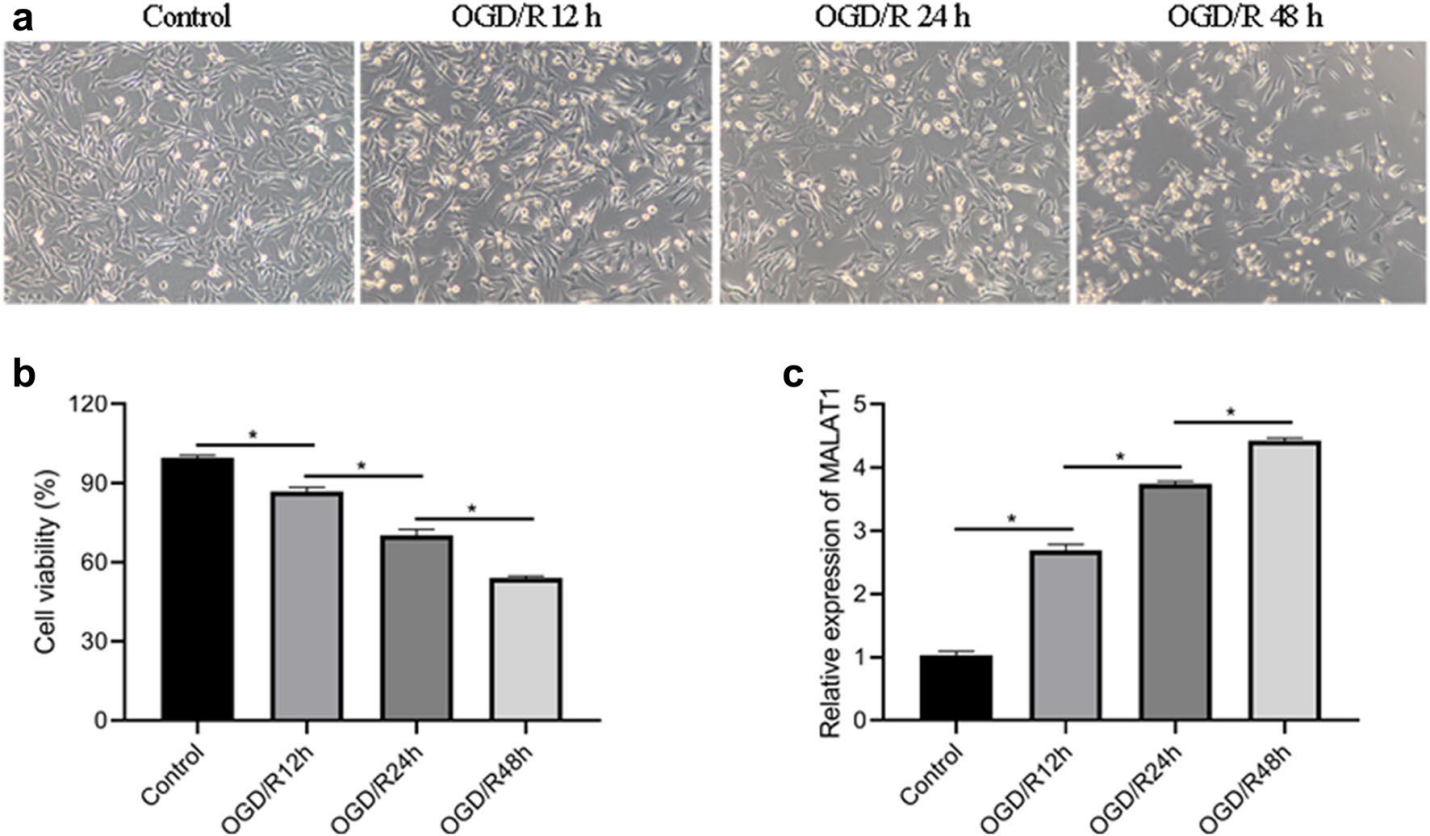
Expression of MALAT1 in OGD/R-induced HT22 cell model. **a** Representative morphology of HT22 cell models. **b** The viability of HT22 cells analysed by CCK-8. **c** Relative MALAT1 mRNA expression in HT22 cell models at 12, 24 and 48 h of reoxygenation. *p < 0.05

The cell model of OGD/R**-**neuronal injury was established by 4 h OGD followed by 12 h, 24 h and 48 h reoxygenation in HT22 neural cells. Cell viability and the expression of MALAT1 were examined at different time points of OGD/R injury. As shown in Fig. 1a, b, cell viability was significantly decreased after 24 h and 48 h of reoxygenation. Moreover, MALAT1 levels were increased in a time-dependent manner, and displayed a significant increase at 48 h post-reoxygenation (Fig. 1c). Thus, the cell model of OGD/R**-**neuronal injury established by 4 h OGD followed by 48 h reoxygenation was chose for further experiments. These findings indicate that upregulated MALAT1 is closely associated with the OGD/R-induced neuronal injury in HT22 neural cells.

### Silenced MALAT1 attenuates OGD/R-induced neuronal injury and ER stress in HT22 cells

**Fig. 2:**
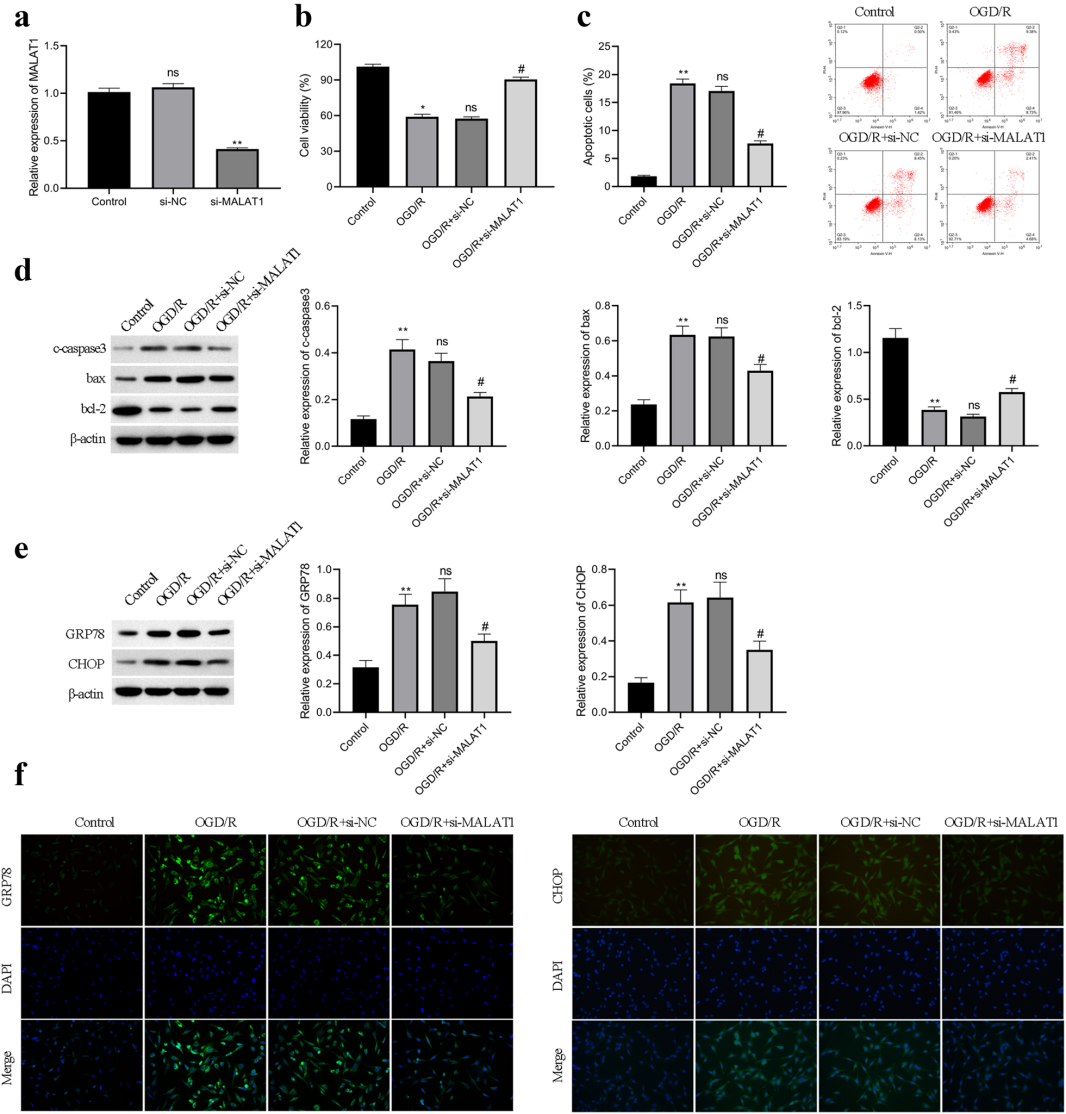
Downregulation of MALAT1 suppressed OGD/R-induced cell injury and ER stress. **a** The expression of MALAT1 in HT22 cell transfected with si-MALAT1 by qRT-PCR. **b** Cell viability as detected by CCK-8. **c** Cell apoptosis was analyzed by flow cytometry. D,Expression of apoptosis-related proteins by Western blot. **e** The expression GRP78 and CHOP proteins by Western blot. *p < 0.05, **p < 0.01 vs. the Control groups; ns: no significance, ^#^p < 0.05 vs. the OGD/R groups

To determine the role of MALAT1 in OGD/R-induced neuronal injury, we used siRNA targeting MALAT1 to inhibit MALAT1 levels in HT22 cells. As shown in Fig. 2a, MALAT1 siRNA could efficiently inhibit MALAT1 levels. CCK8 assay showed that OGD/R significantly decreased the viability of HT22 cells, and MALAT1 siRNA increased cell viability (Fig. 2b). Further, we found that OGD/R significantly increased the apoptosis of HT22 cells, and MALAT1 siRNA reduced the apoptosis of HT22 cells (Fig. 2c), which was also confirmed by western analysis of apoptosis-related markers (Fig. 2d). Moreover, OGD/R significantly increased GRP78 and CHOP expression, and MALAT1 siRNA decreased GRP78 and CHOP expression (Fig. 2e),which was also confirmed by immunofluorescence asssay (Fig. 2f). These observations suggest that MALAT1 promotes neuronal apoptosis and ER stress during OGD/R.

### MALAT1 directly interacts with miR-195a-5p

**Fig. 3:**
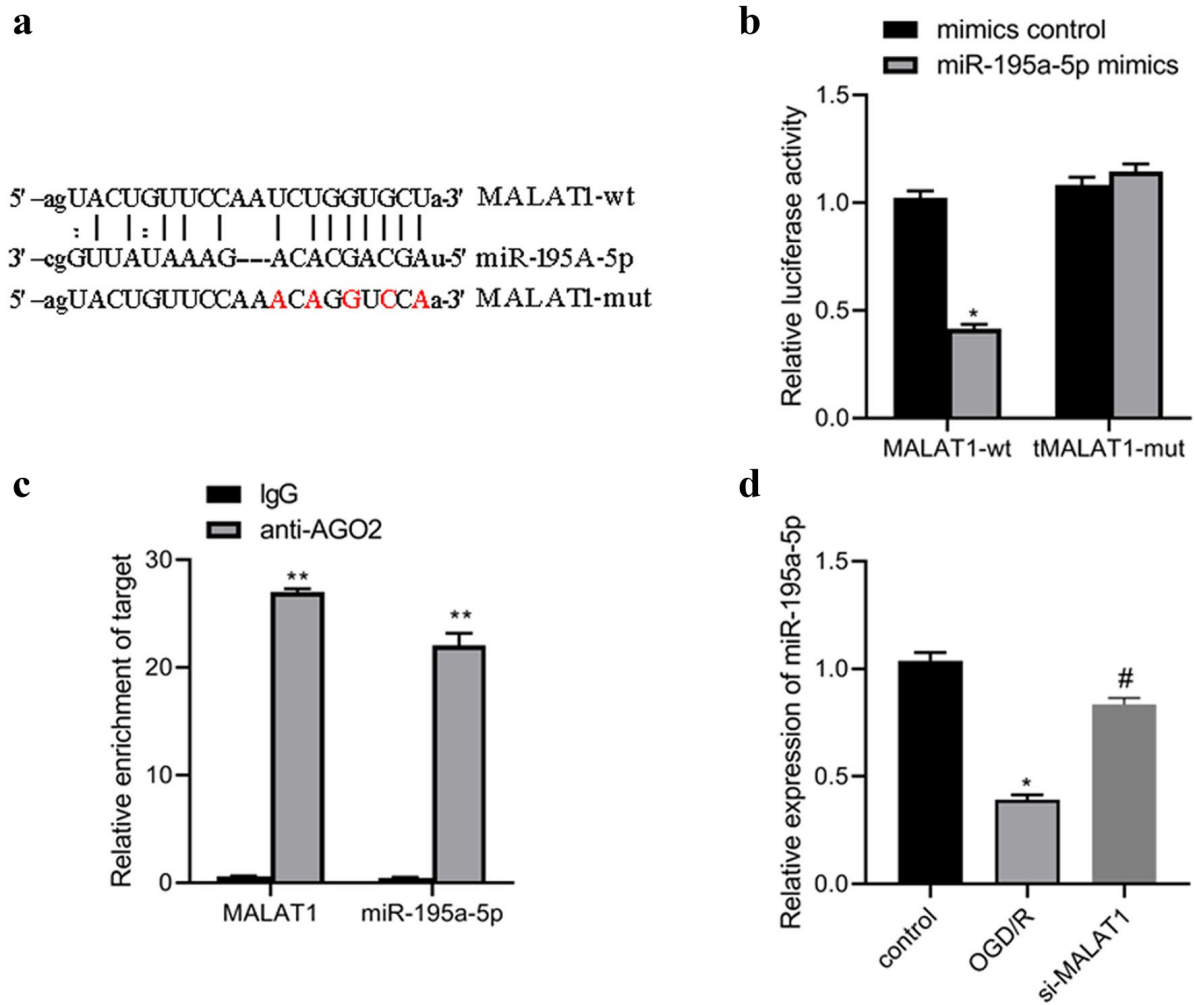
Targeting relationship between miR-195a-5p and MALAT1. **a** Potential binding sites between MALAT1 and miR-195a-5p via RegRNA 2.0. **b** Binding relationship of MALAT1 and miR-195a-5p was confirmed by dual-luciferase reporter gene assay. **c** RIP assay and qPCR detection of MALAT1 binding to miR-195a-5p. **d** Relative mRNA expression of miR-195a-5p in HT22 cells after transfected with MALAT1 siRNA

LncRNAs could compete for binding to miRNAs through miRNA response elements, thus preventing the binding of miRNAs to target mRNA. So we proposed that MALAT1 may function as a ceRNA to regulate miRNAs. Sequence analysis and an open online database starbase () provided putative-binding sites between MALAT1 and miR-195a-5p (Fig. 3a), suggesting its ceRNA potential for miR-195a-5p. Subsequently, we constructed luciferase reporters containing wild type MALAT1 (pmirGLO-MALAT1-wt) or mutant MALAT1 (pmirGLO-MALAT1-mut). We also overexpressed miR-195a-5p via miR-195a-5p mimic. Compared to mimic control, miR-195a-5p mimic remarkably reduced the luciferase reporter activity of the pmirGLO-MALAT1-wt reporter, but had no obvious inhibitory effect on pmirGLO-MALAT1-mut (Fig. 3b), suggesting that MALAT1 is physically associated with miR-195a-5p. To further validate the direct interaction between miR-195a-5p and MALAT1, RIP assay was carried out. We found significant enrichment of miR-195a-5p and MALAT1 using AGO2 antibody than IgG antibody (Fig. 3c). Furthermore, PCR analysis results demonstrated that miR-195a-5p was downregulated in OGD/R treated cells and exhibited a significant increase when OGD/R treated cells were transfected with si-MALAT1 (Fig. 3d). These findings supported that miR-195a-5p was bona fide MALAT1-targeting miRNA.

### miR-195a-5p is essential for MALAT1-mediated neuronal function

**Fig. 4:**
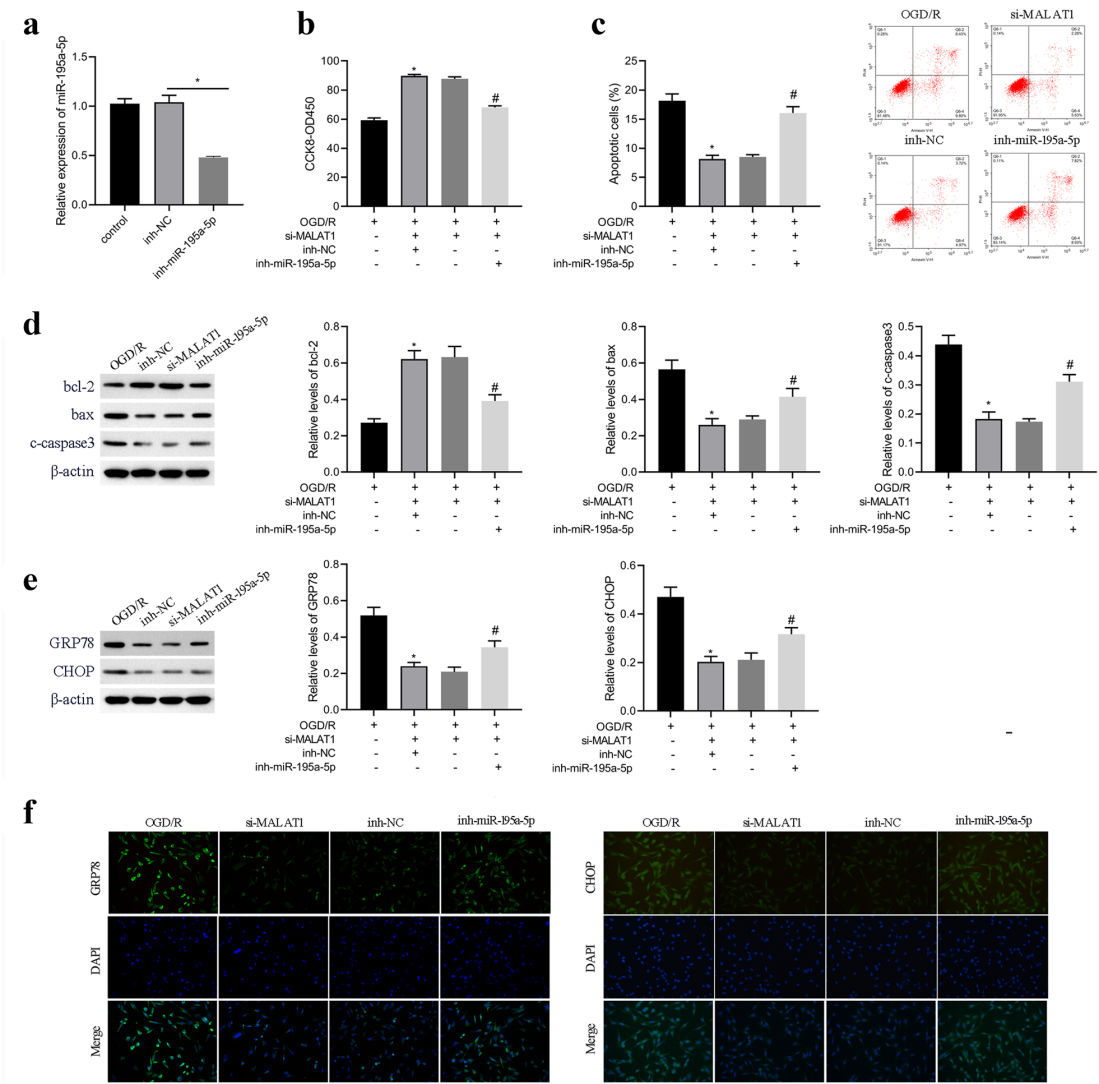
miR-195a-5p is essential for MALAT1-mediated neuronal function. **a** The expression of miR-195a-5p in HT22 cell treated with miR-195a-5p inhibitor by qRT-PCR. HT22 cells under OGD/R condition were treated with si-MALAT1 or cotreated with si-MALAT1 and miR-195a-5p inhibitor, and then subjected to (**b**) CCK-8 assay for cell viability. **c** Flow cytometry analysis for cell apoptosis. **d** Western blot of apoptosis-related proteins. **e**, Western blot of GRP78 and CHOP proteins. *p < 0.05 vs. the OGD/R groups, ^#^p < 0.05 vs. the si-MALAT1 groups

To determine the role of miR-195a-5p in the neurodestructive effect of MALAT1, HT22 cells were transfected with MALAT1 siRNA or miR-195a-5p inhibitor and subjected to CCK8, flow cytometry and western blot analysis. As presented in Fig. 4a, miR-195a-5p inhibitor effectively inhibited the levels of miR-195a-5p. CCK8 assays showed that MALAT1 knockdown promoted cell viability of OGD/R-treated HT22 cells, and miR-195a-5p inhibitor abolished the protective effect of MALAT1 siRNA (Fig. 4b). Moreover, flow cytometry analysis showed that MALAT1 knockdown reduced the OGD/R-induced apoptosis of HT22 cells, and miR-195a-5p inhibitor abolished the inhibition effect of MALAT1 siRNA (Fig. 4c). In addition, western blot analysis showed that MALAT1 knockdown caused decreased caspase-3 and bax and increased bcl-2 protein levels, whereas miR-195a-5p inhibitor abolished the regulatory effect of MALAT1 siRNA on these apoptosis-related proteins (Fig. 4d). Moreover, western blot analysis demonstrated that MALAT1 siRNA downregulated GRP78 and CHOP expression, while miR-195a-5p inhibitor abolished the effect of MALAT1 siRNA (Fig. 4e),which was also confirmed by immunofluorescence asssay (Fig. 4f). These observations suggested that miR-195a-5p is essential for MALAT1-meadited neuron function during OGD/R.

### HMGA1 is the direct target of miR-195a-5p in HT22 cells

**Fig. 5:**
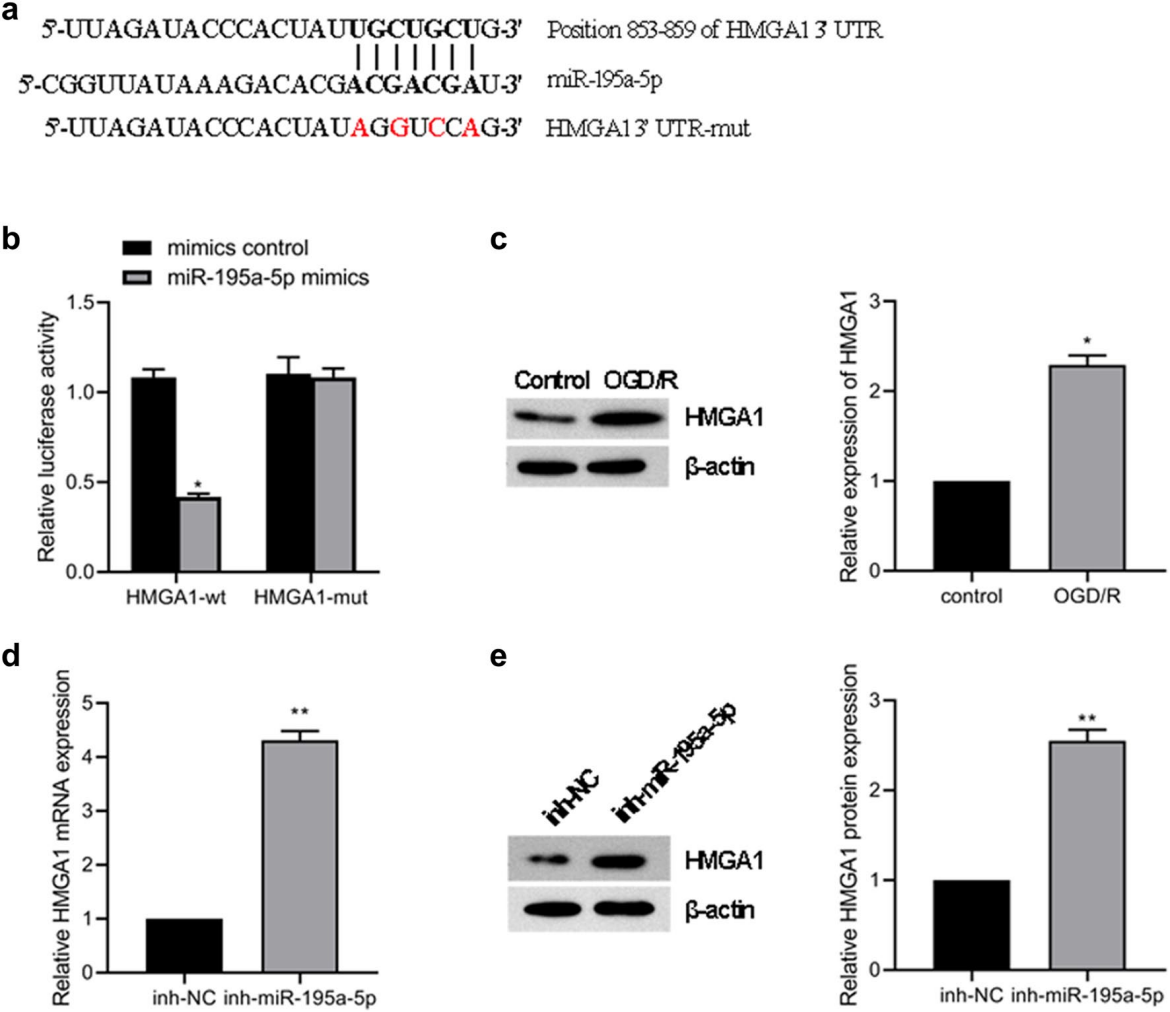
HMGA1 is the downstream target of the MALAT1-miR-195a-5p axis. **a** Putative miR-195a-5p binding sites in HMGA1. The potential complementary residues are shown in red. **b** Relative luciferase activity of wild-type and 3ʹ-UTR mutant constructs of HMGA1 cotransfected with miR-195a-5p mimics and miRNA negative control. ***p < 0.001 versus the miRNA negative control group using one-way ANOVA followed by the Holm-Sidak test. Real-time PCR (**c**) and western blot (**d**) analyses of HMGA1 expression in HT22 cells transduced with miR-195a-5p inhibitor. n = 3. **p < 0.01 and ***p < 0.001 versus the control group using the Student t test (**e**) western blot analyses HMGA1 expression in HT22 cells exposure to OGD/R. **p < 0.01 and ***p < 0.001 versus the control group using the Student t test

To elucidate the molecular mechanisms by which MALAT1-miR-195a-5p regulates cellular function in neural cells, we next searched for candidate miR-195a-5p target genes predicted by TargetScan. The HMGA1 was found to have a conserved miR-195a-5p binding site within its 3ʹ-UTR in most species (Fig. 5a). Cotransfection of miR-195a-5p mimic and a pmiR-GLO plasmid with the wild-type HMGA1 3ʹ- UTR resulted in the downregulation of luciferase activity, and no change was observed in cells transfected with the mutated HMGA1 3ʹ-UTR (Fig. 5b). Moreover, the expression of HMGA1 was upregulated in the OGD/R group compared with that in the control group (Fig. 5c). Moreover, miR-195a-5p inhibitor could increase HMGA1 expression at both the mRNA (Fig. 5d) and protein levels (Fig. 5e) in HT22 cells.

### MALAT1 regulates neuron function via downstream HMGA1

**Fig. 6:**
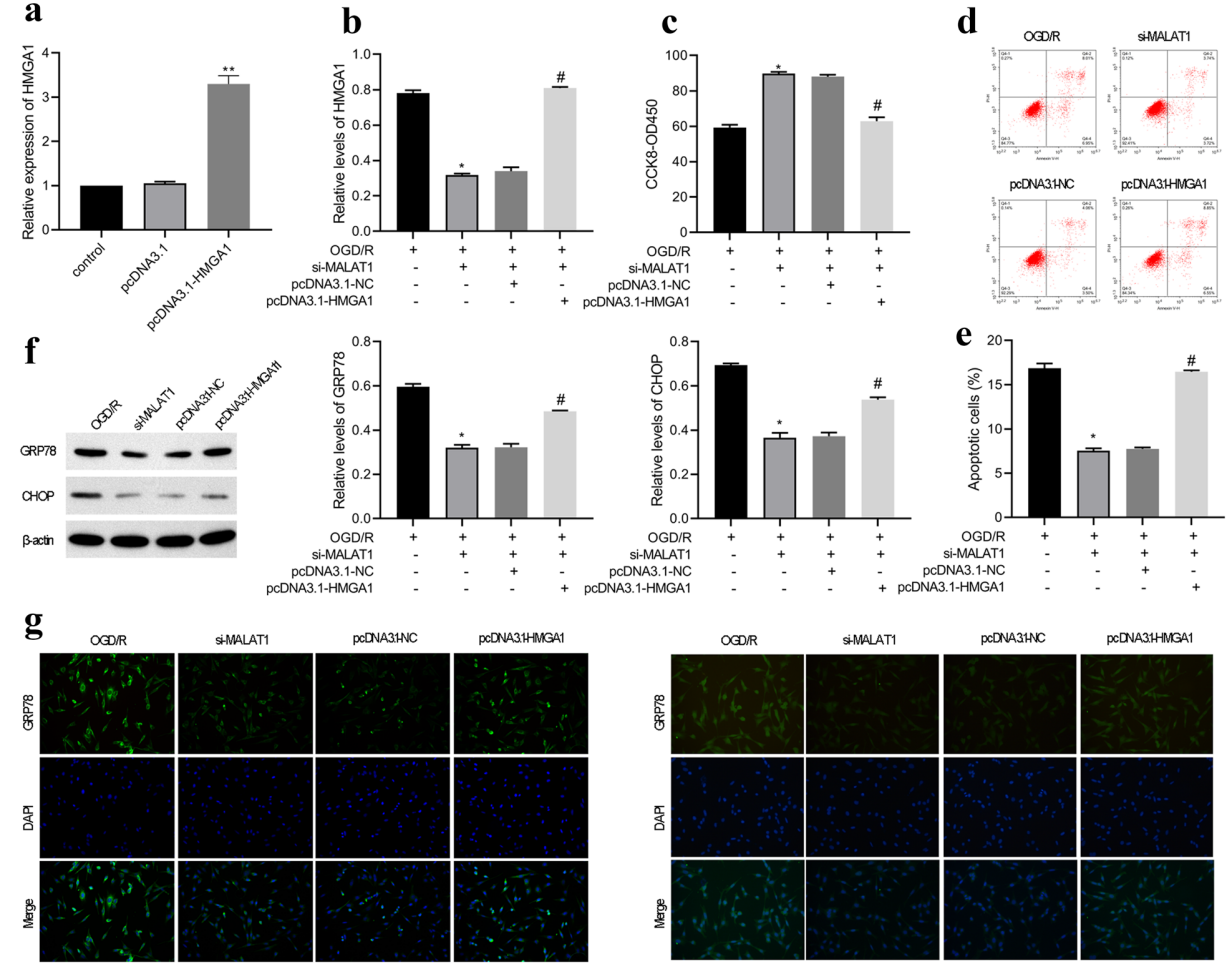
LncRNA-MALAT1 regulates neuron function via its downstream effector HMGA1. **a** Transfection of HT22 cells with pcDNA3.1-HMGA1 significantly promotes the expression of HMGA1 as determined by qRT-PCR. **b** The expression of HMGA1 in HT22 cells, examined by qRT-PCR. **c** Transfection of HT22 cells with pcDNA3.1-HMGA1 significantly abolished the promotion effect of MALAT1 siRNA on cell viability as determined by CCK8 assay. **d, e** Transfection of HT22 cells with pcDNA3.1-HMGA1 significantly abolished the inhibition effect of MALAT1 siRNA on cell apoptosis as determined by flow cytometry analysis. **f** Transfection of HT22 cells with pcDNA3.1-HMGA1 significantly abolished the inhibition effect of MALAT1 siRNA on the expression of GRP78 and CHOP as determined by westen blot analysis. **g** Transfection of HT22 cells with pcDNA3.1-HMGA1 significantly abolished the inhibition effect of MALAT1 siRNA on the expression of GRP78 and CHOP as determined by immunofluorescent staining. n = 3. **p < 0.01 versus the OGD/R groups. ^##^p < 0.01 versus the OGD/R + si-MALAT1 groups

We next sought to examine the role of HMGA1 in MALAT1-mediated neuron function. Transfection with pcDNA3.1-HMGA1 efficiently increased HMGA1 mRNA expression (Fig. 6a). To verify the mechanism of MALAT1 in regulating neuron function, MALAT1 siRNA and pcDNA3.1-HMGA1 or pcDNA3.1-NC were co-transfected into HT22 cells. Then, the HT22 cells were subjected to OGD/R treatment, and the expression of HMGA1 in the modified HT22 cells. Knockdown of MALAT1 remarkably decreased the expression of HMGA1, whereas overexpression of HMGA1 rescued the inhibitory effect of MALAT1 siRNA on HMGA1 expression (Fig. 6b) Further, CCK8 assay showed that MALAT1 siRNA promoted cell viability in HT22 cells treated with OGD/R, which was abolished by HMGA1 overexpression (Fig. 6c). Moreover, MALAT1 siRNA inhibited cell apoptosis in HT22 cells treated with OGD/R, whereas overexpression of HMGA1 abolished the inhibition effect of MALAT1 siRNA (Fig. 6d, e). In addition, overexpression of HMGA1 attenuated the inhibition effect of MALAT1 siRNA on ER stress-related proteins GRP78 and CHOP (Fig. 6f). This finding were also confirmed by immunofluorescent staining (Fig. 6g). Therefore, these results demonstrate that knockdown of MALAT1 promotes viability and inhibits apoptosis of OGD/R-induced HT22 cells by regulating HMGA1.

## Discussion

Ischemia and reperfusion injury causes to tissue damage and destruction, and eventually leads to deterioration and poor prognosis of patients with ischemic stroke [26]. To date, great progress has been made in understanding the mechanisms of reperfusion injury [4]. Increasing evidence has shown that lncRNAs play important roles in the pathogenesis of ischemic and reperfusion injury. In this study, we observed that upregulation of lncRNA MALAT1 expression in OGD/R-induced neuronal injury HT22 cell model. Functional experiments demonstrated that silenced MALAT1 attenuated OGD/R-induced neuronal injury and ER stress in HT22 cells. Mechanically, MALAT1, as an endogenous miR-195a-5p sponge, promotes upregulation of HMGA1, resulting in neuronal injury. Therefore, we identified the adverse effects and mechanism of MALAT1 in ischemia-reperfusion injury.

During both development and adulthood, the human brain expresses many thousands of long lncRNAs, and lncRNAs play important roles in neurodevelopment, neural cell function, and even neurological diseases [27]. Recently, some lncRNAs have been reported to be associated with ischemia reperfusion injury. Zhipeng Xiao et al. [28] reported that repression of H19 alleviates hypoxia/ischemia induced neuronal injury by miR-19a/Id2 axis. Dong You et al. [29] showed that blocking lncRNA MEG3 protects nerve growth and attenuates neurological impairment of rats after cerebral ischemia-reperfusion injury via activating Wnt/β-catenin signaling pathway. Although some lncRNAs have been found in the regulation of ischemic reperfusion injury, these findings are not sufficient to elucidate the complex mechanism. In this study, we found elevated MALAT1 levels in OGD/R-treated neurons, indicating the association between MALAT1 and ischemia reperfusion. To investigate the role of MALAT1, we silenced the expression of MALAT1 and found that knockdown of MALAT1 significantly enhanced the survival ability, alleviated OGD/R-induced apoptosis and ER stress in neurons. These data confirmed the protective effect of MALAT1 silencing on OGD/R-treated neurons by inhibition of neuronal apoptosis and ER stress.

It is well known that ceRNA is a common mechanism of lncRNAs, in which lncRNA upregulates certain mRNAs by absorbing miRNAs. Here, we determined the role of MALAT1 as a molecular sponge of miR-195a-5p in HT22 cells. Functionally, we found that miR-195a-5p is necessary for MALAT1-mediated neurodestructive action. More importantly, HMGA1 was predicted and confirmed to be the target mRNA of miR-195a-5p and regulated by MALAT1 and miR-195a-5p. HMGA1 is a member of the HMG family of architectural transcription factors and involved in chromatin remodeling and regulation of different gene expression depending on the cell context. [30]. HMGA1 is abundantly expressed during embryo development and downregulated in the adult differentiated tissues. Abnormal expression of HMGA1 is closely associated with tumors, cardiovascular diseases, insulin resistance and T2DM, as well as nervous system diseases [31–35]. For neural system, HMGA1 is essential for timing the switching of neural stem-cell competency. The expression of HMGA1 gradually declines with development, which is important for the normal timings of switching from deep-layer neurogenesis to superficial-layer neurogenesis, and from superficial-layer neurogenesis to astrogenesis [36]. HMGA1 can be induced in adult neurons by hypoxia or oxidative stress [37].It is reported that HMGA1 levels were downregulated in the MCAO rats and cortical neurons subjected to OGD, whereas HMGA1 overexpression suppresses cell apoptosis in OGD cortical neurons [38, 39]. Our study showed that HMGA1 was upregulated in OGD/R-treated HT22 cells and contributed to ischemia-reperfusion injury. Besides, further investigates found that HMGA1, regulated by miR-195a-5p, could abrogated MALAT1 siRNA-mediated neuroprotective effect. Together with our findings that MALAT1 contributes to OGD/R-induced neuronal injury via sponging miR-195a-5p to upregulate HMGA1.

## Conclusions

In summary, our study revealed the regulatory mechanism by which lncRNA MALAT1 regulates ischemia-reperfusion injury through targeting the miR-195a-5p/HMGA1 axis. Specific blockage of MALAT1 is expected to be a potential therapeutic target for the protection of neuronal function in stroke patients, which may contribute to the further development of therapeutics against stroke and provide a new opportunity to improve the prognosis of patients.

## Data Availability

The datasets used and/or analysed during the current study are available from the corresponding author on reasonable request.

## Abbreviations

MALAT1: Metastasis associated lung adenocarcinoma transcript 1
OGD/R: Oxygen-glucose deprivation/reoxygenation
HMGA1: High mobility group AT-hook1
ER: Endoplasmic reticulum
GRP78: Glucose-regulated protein 78
CHOP: C/EBP homologous protein
LncRNAs: Long noncoding RNAs
BMEC: Brain microvascular endothelial cells
MCAO: Middle cerebral artery occlusion
qRT-PCR: Quantitative real-time RCR
SDS-PAGE: Sodium dodecyl sulfate-polyacrylamide gel electrophoresis
PVDF: Polyvinylidene fluoride
PI: Propidium iodide
RIP: RNA binding protein immunoprecipitation
SD: Standard deviation
ANOVA: One-way analysis of variance
